# Study on the correlation between microbial communities with physicochemical properties and flavor substances in the Xiasha round of cave-brewed sauce-flavor Baijiu

**DOI:** 10.3389/fmicb.2023.1124817

**Published:** 2023-03-01

**Authors:** Tingting Ren, Wei Su, Yingchun Mu, Qi Qi, Dangwei Zhang

**Affiliations:** ^1^School of Liquor and Food Engineering, Guizhou University, Guiyang, China; ^2^Guizhou Provincial Key Laboratory of Fermentation Engineering and Biological Pharmacy, Guizhou University, Guiyang, China

**Keywords:** cave brewed sauce-flavor Baijiu, Xiasha round, microbial communities, physicochemical properties, volatile flavor substances

## Abstract

The Chishui River basin is the main production area of the sauce-flavor Baijiu. Due to the particularity of sauce-flavor Baijiu technology, a large site of workshops needs to be built for brewing and storage. Therefore, used the natural karst caves of Guizhou province to manufacture the sauce-flavor Baijiu, which has enriched the connotation of sauce-flavor Baijiu and saved valuable land resources. In this study, the fermentation grains in the seven stages during the Xiasha round of the cave-brewed sauce-flavor Baijiu (CBSB) were detected using a combination of physicochemical analysis, Headspace solid-phase microextraction gas chromatography-mass detection, and Illumina HiSeq sequencing methods. The results showed *Unspecified_Leuconostocaceae, Weissella, Unspecified_Bacillaceae, Saccharomycopsis, Thermomyces*, and *Unspecified_Phaffomycetaceae* were the main bacterial and fungal genera in the stacking fermentation (SF). In the cellar fermentation (CF), the *Lactobacillus, Unspecified_Lactobacillaceae, Thermoactinomyces, Saccharomycopsis, Unspecified_Phaffomycetaceae*, and *Wickerhamomyces* were the main bacterial and fungal genera. A total of 72 volatiles were detected in the fermented grains. Linear discriminant analysis Effect Size (LEfSe) identified 23 significantly different volatile metabolites in the fermentation process, including 7 esters, 6 alcohols, 4 acids, 3 phenols, 1 hydrocarbon, and 2 other compounds. Redundancy analysis was used to explore the correlation between dominant microbial genera and physicochemical properties. Starch was the main physicochemical property affecting microbial succession in the SF. Acidity, moisture, and reducing sugar were the main driving factors of microbial succession in the CF. The Pearson correlation coefficient revealed the correlation between dominant microbial genera and significantly different volatile flavor substances. A total of 18 dominant microbial genera were associated with significantly different volatile metabolites, *Lactobacillus, Weissella, Wickerhamomyces*, and *Aspergillus* were shown to play crucial roles in metabolite synthesis. On this basis, a metabolic map of the dominant microbial genera was established. This study provides a theoretical basis for the production and quality control of sauce-flavor Baijiu brewed in natural karst caves and lays a foundation for studying the link between flavor formation and microorganisms.

## 1. Introduction

Distilled spirit has been used in China for over 2,000 years and occupies a very unmatched place in traditional Chinese culture (Liu and Sun, 2018). As one of the most complex and typical Baijiu in traditional Chinese Baijiu, sauce-flavor Baijiu is popular with many consumers (Wang Y. R. et al., 2021). The production process is based on a yearly production cycle, with two feeds, nine steams, eight fermentations, and seven distillations (Wang M. Y. et al., 2018). Due to its unique high-temperature fermentation process and particular environment, sauce-flavor Baijiu is distinguished from others by its complex flavors (Wang et al., 2017). The fermentation of sauce-flavor Baijiu is a complex process involving microbial interactions (Jin et al., 2017) that are influenced mainly by the region they are produced (Bokulich et al., 2014). Sauce-flavored Baijiu relies on unrepeatable ecological resources, and its main production areas are concentrated in Renhuai, Xishui, and Jinsha, of the Guizhou Province, China. Meanwhile, the Chishui River basin is the prime production area of the sauce-flavor Baijiu. The distinct manufacturing process of sauce-flavor Baijiu requires a large area for brewing and storage. The unique natural caves formed by the Guizhou karst terrain can be used for this purpose. Located on the banks of the Chishui River, the Guixian Cave is currently the only natural cave in the country for brewing sauce-flavor Baijiu. The cave's relatively stable temperature and humidity throughout the four seasons provide the unique conditions required for manufacturing cave-brewed sauce-flavor Baijiu (CBSB), thereby giving it more connotations.

The first grain-feeding stage of sauce-flavor Baijiu brewing is called the Xiasha round. Sorghum was gelatinized and decomposed to produce various flavors and precursor substances, which lays a foundation for the follow-up brewing process (Hao et al., 2021). The whole fermentation process is divided into two processes: stacking fermentation (SF) and cellar fermentation (CF). The physical and chemical properties (temperature, moisture, acidity, reducing sugar, starch) of the process are essential factors that affect the evolution of the microbial community (Wang H. et al., 2021). The microorganisms in fermented grains come from high-temperature Daqu and the production environment, including air, soils, water, and production tools (Wang et al., 2019; Zhang H. X. et al., 2021a). Bacteria can use protein and starch and contribute significantly to Baijiu's flavor formation (Zuo et al., 2020; Tu et al., 2022). Yeast mainly produces various flavor compounds and alcohol (Hu et al., 2020). Molds can secrete different enzymes to decompose raw materials and provide energy for the growth of other bacterial groups (Wang et al., 2014; Hu Y. L. et al., 2021).

Recent research on Chinese Baijiu has focused on the succession of microbial communities, the screening of functional microorganisms, and the correlation between the microbial communities and the physicochemical properties and flavor substances (Zou et al., 2018a; Jin et al., 2019; Song et al., 2019; Mu et al., 2022). These studies have revealed the core functional microorganisms of the fermentation process of Chinese Baijiu and their contribution to the flavor substances, laying a solid foundation for Chinese Baijiu research. However, these studies are all performed on the fermentation process in a conventional environment distillery, and rare research on brewing Baijiu in natural caves has been reported.

Physicochemical analysis, high-throughput sequencing, and headspace solid-phase micro-extraction gas chromatography-mass spectrometry (HS-SPME-GC-MS) were carried out to analyze the physicochemical properties, the microbial community structure, and the volatile flavor substances. In addition, redundancy analysis (RDA) and the Pearson coefficient analysis were performed to assess the correlation between microbiota and physicochemical properties with the volatile flavor substances. The results enabled metabolic mapping of the dominant genera during the Xiasha round of CBSB. This study can provide a theoretical basis for regulating the CBSB fermentation process.

## 2. Materials and methods

### 2.1. Sample collection

**Figure 1:**
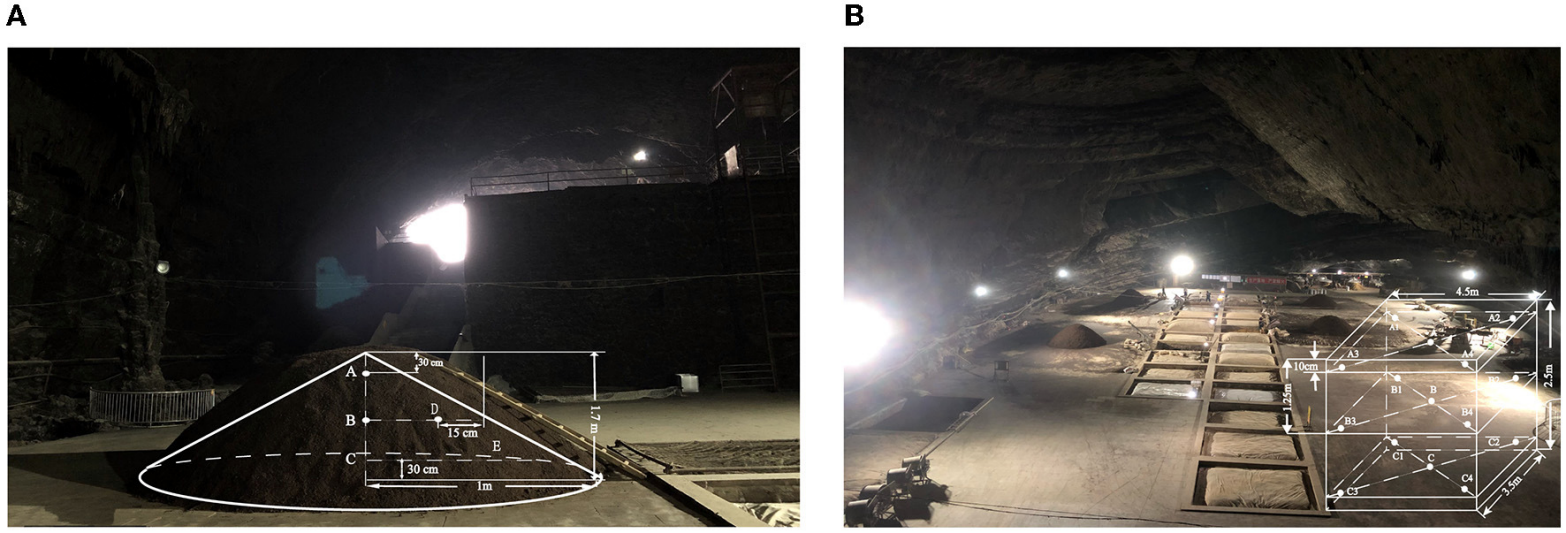
Spoil sampling points **(A)** sketch map of stacking fermentation sampling; **(B)** sketch map of cellar fermentation sampling.

The fermented grain samples were manufactured between September 2020 and October 2020 during the Xiasha round at the Dongniangdongcang Winery, Guizhou Province, China. The fermentation process was carried out in two distinct phases: SF and CF. Samples of SF were collected on days 0, 1, 2, and 3 and marked as SF0, SF1, SF2, and SF3, respectively. For the CF stage, the samples were collected at 10-day intervals until the end of fermentation and marked as CF10, CF20, and CF30. The SF and CF sampling diagrams are shown in Figures 1A, B, respectively. The samples were collected from the upper, middle, and bottom layers of the fermentation piles and cellars at each sampling time point. One point was selected in the middle and four points at the edge of each layer, and at each point, 30 g of samples was collected. All samples collected from each point of all three layers were then evenly combined into one mixture to eliminate sampling error (Zhao et al., 2021). After mixing, they were immediately packed in a sterile sealed bag and stored in a low-temperature refrigerator at −80°C until analysis.

### 2.2. Analysis of physicochemical indicators

To understand the fermentation processes, temperature, moisture, acidity, reducing sugar, and starch were detected. An alcohol thermometer was inserted into the fermented grains using a self-made tool, and the temperature was measured quickly after 5 min. The moisture of fermented grains was determined by a gravimetric method by drying samples to a constant weight at 105°C. The titratable acidity was determined by titration with a standard NaOH (0.1 mol/L) solution. A standard glucose solution (1.0 g/L) was used to titrate reducing sugar and starch in the fermented grains.

### 2.3. Analysis of volatile flavor substances

The fermented grain sample was ground and mixed, and 1.00 g of it was placed in a 20 ml headspace flask with 2 g of NaCl and 5 ml of purified water. Then, 10 μl of cyclohexanone (20 μg/ml) was added as the internal standard. The SPME fibers were extracted by 50/30 μm DVB/CAR/PDMS fiber (Sigma Aldrich, USA) at 40°C for 180 min, inserted into the injection port, and then separated by resolution at 230°C for 5 min.

Chromatographic conditions: A gas chromatography-mass spectrometer was used to analyze the fermented grain (Thermo Fisher Scientific, USA). The capillary column was DB-5MS (30 m × 0.25 mm × 0.25 μm, Agilent, USA) with helium (99.999%) as the carrier gas at a 1.0 ml/min flow rate in the non-split mode. Heating procedure: The column was maintained at 40°C for 5 min, then increased to 150°C at 5°C/min, later maintained at 150°C for 3 min, and finally increased to 240°C at 5°C/min and maintained for 5 min. Mass spectrometry conditions: electron bombardment ion source (EI), electron energy of 70 eV, a transmission line temperature of 280°C, and an ion source temperature of 230°C. The data were collected in the 50–450 amu at a rate of 1 scan/s. The constituents were tentatively identified by matching the mass spectrum with the NIST5 spectrum database and comparing their Kováts retention index (RI) with the RI reported in the literature, calculated using the C7–C40 n-alkanes, for verification.

### 2.4. Analysis of the microbial community

Total genomic DNA was extracted with the FastDNA^®^ Spin Kit according to the manufacturer's protocol. DNA purity and concentration were checked using a NanoDrop 2000 ultra-micro spectrophotometer, followed by 1% agarose gel electrophoresis at 5 V/cm for 20 min to check DNA integrity. The V3-V4 region of the bacterial 16S rRNA gene was amplified with primers 338F (5′-ACTCCTACGGGAGGCAGCAG-3′) and 806R (5′-GGACTACHVGGGTWTCTAAT-3′), and the ITS1 region of the fungi was amplified with the primers ITS1F (5′- CTTGGTCATTTAGAGGAAGTAA-3′) and ITS1R (5′-GCTGCGTTCTTCATCGATGC-3′).

The total PCR amplification volume was 20 μl, including 0.8 μl of the forward primer (5 μm), 0.8 μl of the reverse primer (5 μm), 2 μl of 2.5 mm dNTPs, 2 μl of 10 × buffer, 0.2 μl of rTaq polymerase, 0.2 μl of BSA, 10 ng of template DNA, and 20 μl of ddH_2_O. The PCR conditions were as follows: initial denaturation at 95°C for 3 min, denaturation at 95°C for 30 s, annealing at 53°C and 55°C for 30 s for bacteria and fungi, respectively, followed by extension at 72°C for 45 s, 29 and 35 cycles for bacteria and fungi, respectively, and final extension at 72°C for 10 min and cooling to 10°C to end the reaction. All PCR products were tested for concentration and purity using NanoDrop2000, and 2% agarose gel electrophoresis was used for integrity testing.

The libraries were quantified and tested by Qubit. Then, the purified amplicons were pooled in equimolar amounts and paired-end sequenced (2 × 300) by using an Illumina MiSeq platform (Illumina, San Diego, USA) according to the standard protocols. Each sample was quality filtered, trimmed, denoised, and merged. Then, the chimeric sequences were identified and removed using the QIIME2 dada2 plugin to obtain the feature table of the amplicon sequence variant (ASV) (Callahan et al., 2016). Taxonomic annotation was carried out on the obtained ASVs based on the SILVA (bacteria) and UNITE (fungi) taxonomic databases.

### 2.5. Statistical analysis

The experiments were repeated three times; data analyses were performed using IBM SPSS statistics 26.0, and the obtained data were analyzed by ANOVA with a *P-*value of < 0.05 as the significance level. A principal component analysis was performed using SIMCA (version 14.1), and the graphs were drawn using Origin (version 9.8). Visualized correlation data painting was performed using the Cytoscape software (version 3.8.2).

## 3. Results and analysis

### 3.1. Changes in the physicochemical properties

As shown in Table 1, due to the enrichment and rapid growth of microorganisms, the temperature reached a maximum of 45.67 ± 8.02°C on day 3 of the SF process. We observed the time required by the sample to reach the highest temperature in the SF stage and found that it was consistent with that of the sauce-flavor Baijiu brewed in a conventional environment distillery (Hao et al., 2021). The acidity increased significantly (*P* < 0.05) on day 3 of SF, and then, the increase was more significant in the CF stage, which may be due to the accumulation of acid produced by the microorganisms during anaerobic fermentation (Cai et al., 2019). The starch content decreased significantly (*P* < 0.05) on day 1 of SF from 40.96 ± 0.04% to 39.24 ± 0.30%, with a decreasing trend in the CF stage. The reduced sugar content increased significantly (*P* < 0.05) on day 2 in the SF stage and significantly decreased in the CF stage (*P* < 0.05). The rapid growth and multiplication of microorganisms in the SF stage consumed starch to produce monosaccharides, which were further used to produce more flavors during the CF stage (Wu et al., 2013; Hu X. L. et al., 2021). The growth and reproduction of microorganisms produced a large amount of biological heat (Cai et al., 2019). Therefore, the moisture content tended to decrease during the SF process. The increased moisture content during CF was related to the interaction between microorganisms (Huang et al., 2020; Hu Y. L. et al., 2021).

**Table 1:** Changes in the physicochemical properties during the fermentation process.

**Samples**	**Temperature (°C)**	**Moisture (%)**	**Acidity (ml/g)**	**Reducing sugar (in glucose, %)**	**Starch (%)**
SF0	27.00 ± 5.00^b^	41.53 ± 0.15^e^	0.55 ± 0.08^e^	0.12 ± 0.01^e^	40.96 ± 0.04^a^
SF1	27.33 ± 5.13^b^	41.46 ± 0.15^e^	0.57 ± 0.03^e^	0.13 ± 0.01^e^	39.24 ± 0.30^b^
SF2	38.00 ± 8.66^ab^	40.71 ± 0.27^f^	0.65 ± 0.04^e^	0.55 ± 0.03^c^	38.65 ± 0.03^c^
SF3	45.67 ± 8.02^a^	43.41 ± 0.26^d^	0.83 ± 0.05^d^	0.82 ± 0.01^a^	38.45 ± 0.22^c^
CF10	31.33 ± 3.22^b^	45.75 ± 0.28^b^	1.81 ± 0.00^c^	0.64 ± 0.02^b^	36.59 ± 0.28^d^
CF20	31.33 ± 3.22^b^	45.01 ± 0.08^c^	2.48 ± 0.07^b^	0.57 ± 0.03^c^	35.79 ± 0.03^e^
CF30	30.33 ± 4.16^b^	46.30 ± 0.09^a^	2.77 ± 0.05^a^	0.50 ± 0.01^d^	32.52 ± 0.03^f^

Different letters in the same column indicate significant differences (*P* < 0.05).

### 3.2. Differences in the microbial community structure during the fermentation process

The changes in the microbial community structure of the 21 samples from 7 time points were detected. The effective sequences of the bacteria and fungi ranged from 23,824 to 38,841 and 16,487 to 39,450, respectively. Although the number of fungal sequences in the samples was greater than that of bacteria, the total number of bacterial ASV was much greater than that of fungi (Supplementary Table S1). In addition, the sparsity curve reached a saturation plateau, indicating that the sequencing depth can represent the microbial diversity of the samples (Supplementary Figure S1). The samples were analyzed for α-diversity, and Supplementary Table S2 lists the species richness and diversity parameters, including the Chao1, Shannon, and Simpson indices. The diversity indices were consistent with the ASV results. Bacterial richness was greater than fungi in the SF, while the opposite was true in the CF stage. During the CF stage, bacterial diversity decreased but fungal diversity increased.

**Figure 2:**
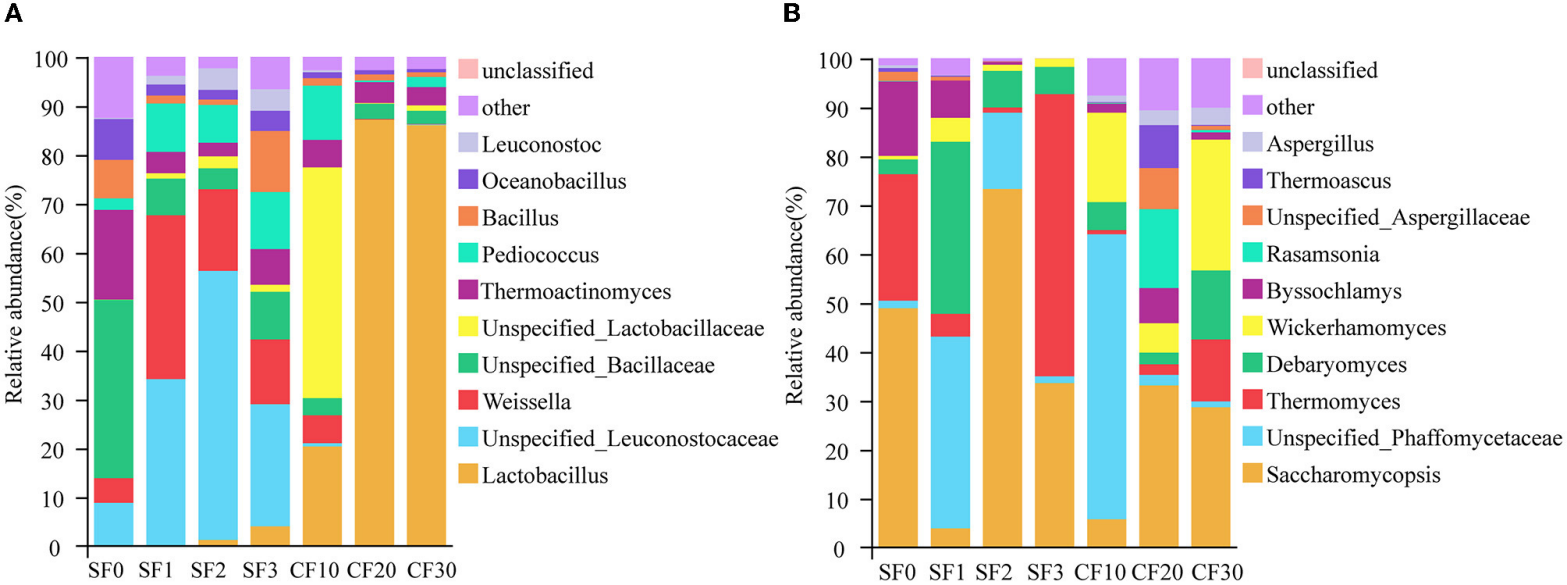
Relative abundance of bacterial **(A)** and fungal **(B)** genera in the Xiasha round of the cave-brew sauce-flavor Baijiu.

The results showed that, among 19 bacterial phyla and 185 bacterial genera, Firmicutes was the dominant bacterial phylum (90.18–99.73%). We defined the top 10 genera in relative abundance as the dominant microorganism genera to study community succession during fermentation (Figure 2). In all samples, the dominant bacterial genus contributed to 95.28% of the bacterial sequences, as shown in Figure 2A. The dominant microbial genera include *Lactobacillus* (28.46%), *Unspecified_Leuconostocaceae* (17.63%), *Weissella* (10.65%), *Unspecified_Bacillaceae* (9.60%), *Unspecified_Lactobacillaceae* (7.63%), *Thermoactinomyces* (6.64%), *Pediococcus* (6.43%), *Bacillus* (3.83%), *Oceanobacillus* (2.79%), and *Leuconostoc* (1.62%).

The bacterial community in the fermented grains changed significantly during the fermentation process (Figure 2A). *Unspecified_Bacillaceae* and *Thermoactinomyces* were the dominant genera during the SF1, which might be derived from high-temperature Daqu (Zuo et al., 2020). As SF proceeded, *Unspecified_Leuconostocaceae* and *Weissella* gradually replaced *Unspecified_Bacillaceae* and *Thermoactinomyces* as the dominant genera between days 1 and 3. The reason for the change in *Weissella* abundance may be due to the floor, tools, etc., in the operating environment (Wang X. S. et al., 2018). After the end of SF and into the cellars, which was anaerobic fermentation, the dominant bacterial genera were *Lactobacillus, Unspecified_Lactobacillaceae*, and *Pediococcus* at day 10 of CF. These acid-tolerant microorganisms gradually replaced the non-acid-tolerant ones as the acidity of the anaerobic fermentation system increased. During days 20–30 of CF, *Lactobacillus* became the dominant microbial genus. *Lactobacillus* can produce lactic acid and a variety of antimicrobial substances to inhibit the growth of pathogens and microorganism spoilage during brewing (Galati et al., 2014). Therefore, the abundance of some non-acid-tolerant and pathogenic bacteria, such as *Enterococcus* spp. and *Klebsiella* spp., was gradually decreased as *Lactobacillus* became the dominant bacteria genus during the fermentation process.

In the previous study of the conventional environment distillery, Wang et al. (2015) found that the main bacterial groups of sauce-flavor Baijiu SF and CF were *Bacillaceae* and *Lactobacillales*. In this study, we found that the dominant bacteria family was *Leuconostocaceae*, which was different from the conventional environment distillery of the SF. However, in the anaerobic fermentation, the dominant bacteria order also was *Lactobacillales*, which was the same as the conventional environment distillery in the CF. This indicates that, with the change in the environment, there is a change in the dominant microorganisms in the SF but has little impact on the CF.

A total of six fungal phyla and 102 fungal genera were identified, and Ascomycota was the dominant phylum (96.80–100.00%). Meanwhile, *Saccharomycopsis* (32.44%), *Unspecified_Phaffomycetaceae* (17.05%), *Thermomyces* (15.00%), *Debaryomyces* (10.52%), *Wickerhamomyces* (8.51%), *Byssochlamys* (4.86%), *Rasamsonia* (2.40%), *Unspecified_Aspergillaceae* (1.74%), *Thermoascus* (1.44%), and *Aspergillus* (1.22%) were the dominant fungal genera. During the SF, *Saccharomycopsis, Thermomyces, Unspecified_Phaffomycetaceae*, and *Debaryomyces* were predominant, accounting for 89.49% of the total abundance. The CF's main genera were *Saccharomycopsis, Wickerhamomyces*, and *Unspecified_Phaffomycetaceae*, accounting for 60.01% of the total abundance. The dominant fungi during SF and CF of sauce-flavor Baijiu in conventional environments were *Pichia, Saccharomyces*, and *unidentified* (Hao et al., 2021). The Xiasha round is the non-wine-producing round in the production cycle of CBSB. The role of microorganisms is mainly focused on the process of aroma production and precursor substance production (Wang M. Y. et al., 2018). For example, *Saccharomycopsis*, which is more abundant, although it is not a significant brewing yeast, produces a variety of enzymes to break down starch and is vital in terms of flavor determination and aroma complexity of the final product (Padilla et al., 2016; Boro et al., 2022). Wang H. et al. (2021) analyzed microbiome diversity and evolution during SF for seven rounds in conventional environment distilleries and found that the dominant fungal were *Thermomyces, Byssochlamys, Thermoascus, unclassified_o_Eurotiales*, and *Aspergillus*. Previous studies showed that the fungi in fermentation mainly come from the ground of the fermentation environment (Zhang H. X. et al., 2021a). Therefore, we suppose that the environment is the most crucial factor that affects the fungal community structure in the fermentation process of sauce-flavor Baijiu.

The community structure of microorganisms is closely related to the interactions between microorganisms (Wang Y. R. et al., 2021). Pearson correlation coefficients and *p*-values were calculated to evaluate the interactions at the microbial genus level whose relative abundance was >1%. We found 20 nodes and 33 edges (Supplementary Figure S2). The results of the correlation analysis between bacterial genera showed that *Lactobacillus* was significantly negatively correlated with *Oceanobacillus* and *Unspecified_Leuconostocaceae* (*P* < 0.01, r < −0.5). *Lactobacillus* is often found as the dominant bacteria in Baijiu fermentation, which has a regulatory function on the microbial flora in the Baijiu fermentation system and has a positive role in the production of Baijiu flavor substances (Cai et al., 2021; Wang H. et al., 2021). It can produce acetic acid by heteromorphic lactic acid fermentation during anaerobic metabolism (Sifeeldein et al., 2018). *Bacillus* was significantly positively correlated with *Thermoactinomyces, Saccharopolyspora, Oceanobacillus*, and U*nspecified_Bacillaceae* (*P* < 0.05, r > 0.5). *Bacillus* was identified as the dominant bacteria in the previous studies on the sauce-flavor Baijiu (Hao et al., 2021; Zhang H. X. et al., 2021b). In this study, it is not surprising that the average relative abundance of *Bacillus* has yet reached the maximum at the end of the SF. *Bacillus* can secrete various enzymes to degrade the fermentation substrate to meet its growth and produce Baijiu flavor substances (Cai et al., 2021; Xiao et al., 2021). The correlation results between fungi showed that *Aspergillus* was significantly positively correlated with *Wickerhamomyces* (*P* < 0.05, r > 0.5). In Baijiu fermentation, *Aspergillus* is a functional microorganism with a good saccharification effect (Wu et al., 2021) and is critical for flavor compound production (Jin et al., 2019; Liu et al., 2021). *Saccharomycopsis* was significantly negatively correlated with *Unspecified_Phaffomycetaceae* (*P* < 0.01, r < −0.5). *Saccharomycopsis* was the dominant fungal genus in high-temperature Daqu and plays an essential role in the formation of the unique flavor of Baijiu (Jiang et al., 2021; Zeng et al., 2022).

The results of the correlation between bacteria and fungi showed that *Wickerhamomyces* and *Aspergillus* were significantly positively correlated with *Lactobacillus* (*P* < 0.01, r > 0.5). *Wickerhamomyces* and *Aspergillus* can produce multiple enzymes to decompose raw materials and create conditions for the rapid growth of *Lactobacillus*. *Thermomyces* was significantly positively correlated with *Saccharopolyspora* and *Bacillus* (*P* < 0.05, r > 0.5). It can survive high temperatures and produce enzymatic decomposition materials (Liu and Miao, 2020). *Saccharopolyspora* and *Bacillus* can produce important biologically active substances to make the flavor more complex (Jiang et al., 2021). *Aspergillus* was significantly negatively correlated with *Weissella* and *Unspecified_Leuconostocaceae* (*P* < 0.01, r < −0.5), indicating that *Aspergillus* had an antagonistic effect on both. The flavor and quality of Baijiu can be affected by microbial interaction and the regulation of microbial structure (Zou et al., 2018b; Gao et al., 2022). In summary, these microorganisms coordinate and control each other during the fermentation process in the Xiasha round of CBSB. These mutual biological relationships constitute the primary biological control mechanism of the fermentation process of the CBSB, which is also the fundamental component of flavor formation.

### 3.3. Dynamic changes in flavor substances in fermented grains

**Figure 3:**
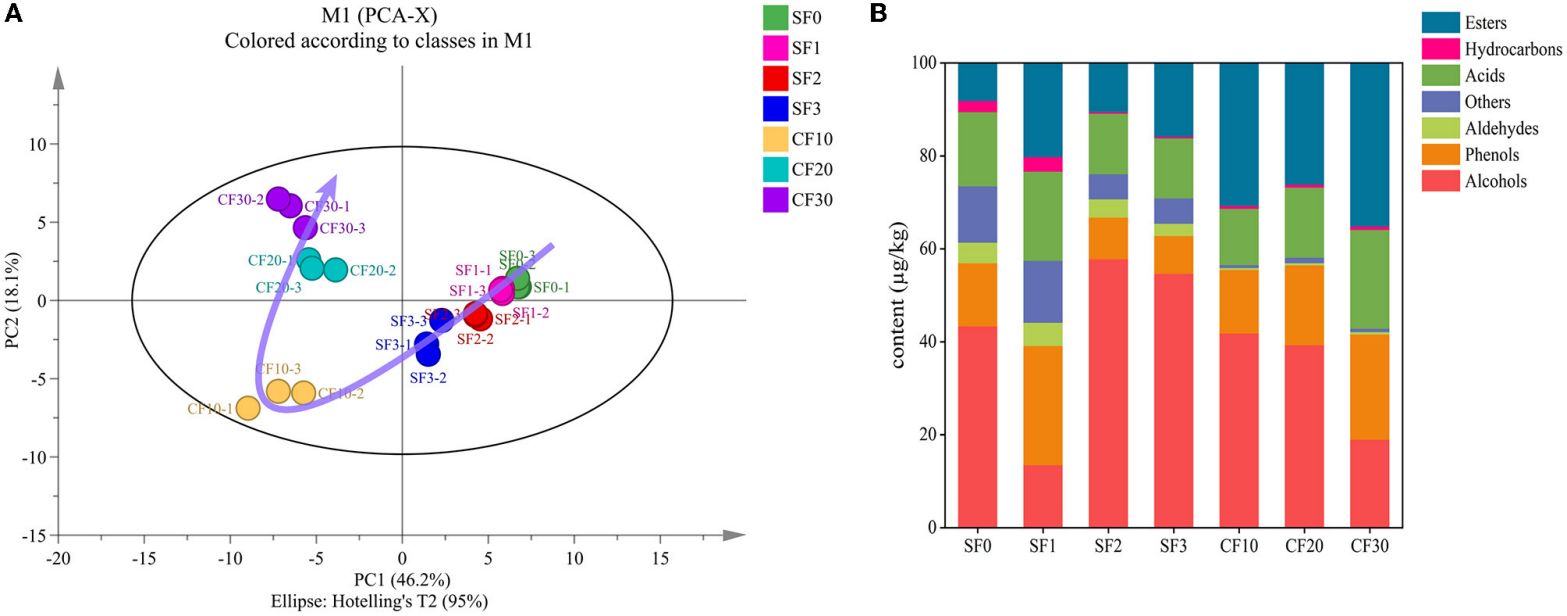
Principal component analysis of volatile compounds **(A)** and percent stacked column chart of volatile flavor substances **(B)**.

In total, 72 volatile flavor substances were detected, including 23 esters, 13 alcohols, 11 acids, 8 phenols, 6 hydrocarbons, 4 aldehydes, and 7 other compounds (Supplementary Table S3). As shown in Figure 3A, the clusters on days 0, 1, 2, and 3 in SF were clearly different from those on day 10 of CF. This result is mainly due to changes in the oxygen level of the fermentation environment that eventually led to changes in the microbiota (Figure 2) and rapid changes in the metabolites in the fermented grains. Moreover, in the CF stage, the fermentation progressed very rapidly for the first 20 days and then changed slowly. This rapid metabolic change may be related to the favorable temperature and sufficient oxygen content available during CF for the first 20 days.

The changes in the contents of volatile compounds during fermentation are shown in Figure 3B and Supplementary Table S4. Esters, acids, and phenols showed an increasing trend during the SF stage. Meanwhile, the alcohol content decreased significantly on day 1, which is closely related to the increase in other flavor substances. During SF, the alcohol content increased with the extension of fermentation time (Wei et al., 2020). While the high phenol content in the early period may be associated with the raw material sorghum (Rao et al., 2018; Li et al., 2021), the higher content of esters in the later period might be related to the accumulation of ester precursors and the production of metabolites by microorganisms (Xu et al., 2022). The significant increase in alcohol content in the CF10 might be caused by the rapid growth and reproduction of alcohol-producing yeast such as *Saccharomyces* (Cai et al., 2019). As oxygen depletion and the available substances inhibited the production of alcohol, the alcohol content decreases significantly. With the rapid growth of acid-producing microorganisms, the growth of ester-producing microorganisms is inhibited (Wu et al., 2017). Therefore, the content of esters decreased significantly in CF20. The contents of esters, phenols, and aldehydes were increased in CF30, which might be due to the transformation of alcohols into other substances by microorganisms and enzymes (Dai et al., 2020; Hong et al., 2020).

**Figure 4:**
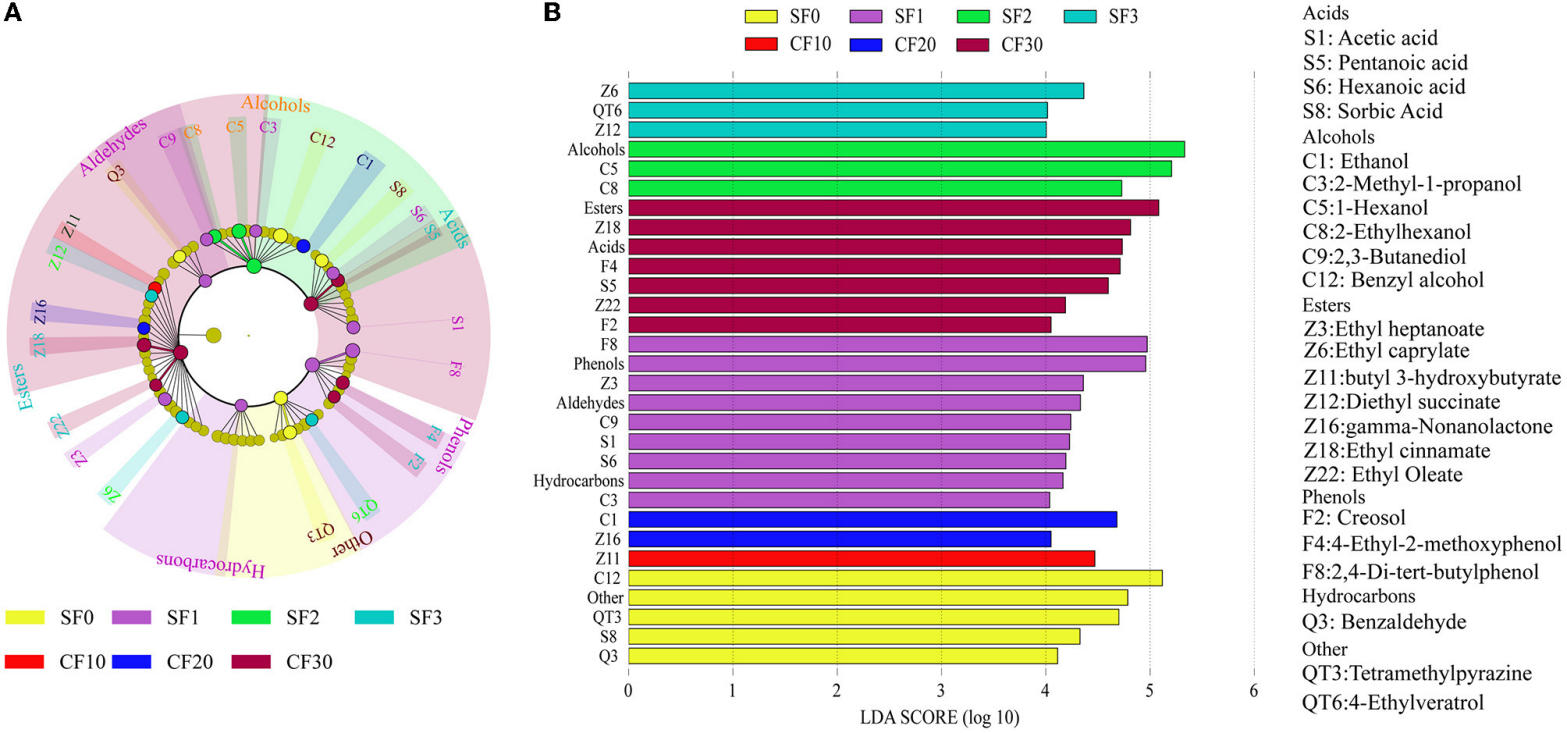
The LEfSe analysis of the flavor components in the Xiasha round of cave-brew sauce-flavor Baijiu. Cladogram **(A)** and LDA bar graph **(B)** results for flavor metabolite contents. Flavor compounds were evaluated by linear discriminant analysis coupled with effect size (LDA = 4, *P* < 0.05).

LEfSe analysis was performed to identify significantly different metabolites in each stage in the Xiasha round of the CBSB. A total of 23 compounds were significantly different in the Xiasha round of the CBSB (LDA = 4, *P* < 0.05) (Figure 4).

Ethyl heptanoate (Z3), ethyl caprylate (Z6), butyl 3-hydroxybutyrate (Z11), diethyl succinate (Z12), gamma-nonalactone (Z16), ethyl cinnamate (Z18), and ethyl oleate (Z22) were the differential eaters (Figure 4A). Among them, diethyl succinate, gamma-nonalactone, and ethyl oleate were detected throughout the fermentation process. Esters in Baijiu were usually synthesized by microbial metabolic pathways, with a small percentage coming from environmental and spontaneous chemical reactions (Xu et al., 2021). The levels of ethyl heptanoate, ethyl octanoate, and ethyl cinnamate gradually increased with increasing fermentation time during the Xiasha round, reaching a maximum on day 10 of CF and then decreased. Butyl 3-hydroxybutyrate was detected on day 0 and day 1 of SF and on days 20 and 30 of CF, which was not detected the rest of the time. Among them, ethyl heptanoate, ethyl octanoate, and ethyl cinnamate were not detected in the SF0, and their content increased with fermentation. This was sufficient to prove that these three esters were produced by microbial metabolism and esterification during the fermentation process (Dong et al., 2022). Moreover, because of the high content of ethanol produced during the fermentation of Chinese Baijiu, ethanol and acid undergo biochemical reactions to produce esters, which may also account for the great variety and concentration of ethyl esters. It can add fruit and floral aromas to Baijiu and make Baijiu taste soft, sweet, and astringent (Xu et al., 2022).

Fatty acids are crucial for the flavor of Chinese Baijiu, and microorganisms can synthesize low-molecular-weight fatty acids such as butyric, valeric, hexanoic acid, and octanoic acids, which are precursors of ester compounds (Wei et al., 2020). During the Xiasha round of CBSB, acetic acid (S1), pentanoic acid (S5), hexanoic acid (S6), and sorbic acid (S8) are the differential metabolites (Figure 4). While acetic acid and hexanoic acid were detected throughout the fermentation process, pentanoic acid was detected only on day 2 of SF and on day 10 of CF. In contrast, sorbic acid was detected only at the beginning and end of fermentation. Strong-flavored Baijiu is rich in hexanoic acid, which is a volatile flavoring substance that contributes much to the flavor (Fan et al., 2019). The acetic acid content was the highest throughout the fermentation stage. Although acetic acid produced a pungent odor, it could increase the flavor of Chinese Baijiu upon its combination with alcohol to produce esters, such as ethyl acetate (Cai et al., 2019).

Alcohols are auxiliary agents that coordinate Baijiu aroma and taste (Zhang M. Z. et al., 2021). In the differential alcohol metabolites during the Xiasha round of CBSB (Figure 4), the ethanol content showed an increasing trend during SF and CF, and it is an essential indicator of the fermentation performance of Baijiu (Wei et al., 2021). Furthermore, the higher alcohols 2-methyl-1-propanol (C3), benzyl alcohol (C12), 1-hexanol (C5), 2,3-butanediol (C9), and 2-ethylhexanol (C8) are present in many alcoholic beverages. Moderate amounts of higher alcohols increase the flavor and taste of Baijiu (Han et al., 2014).

Creosol (F2), 4-ethyl-2-methoxyphenol (F4), 2,4-di-tert-butylphenol (F8), tetramethylpyrazine (QT3), 4-ethylveratrol (QT6), and benzaldehyde (Q3) were also differential metabolites found during the Xiasha round of CBSB (Figure 4A). Creosol, 4-ethyl-2 methoxyphenol, and 2,4-di-tert-butylphenol showed an increasing trend during fermentation, while tetramethylpyrazine showed a decreasing trend. Benzaldehyde was detected throughout the fermentation stages, whereas 4-ethylveratrol was detected only in a few fermentation stages. Although these substances are a small part of the fermentation process, like phenolics, they are antioxidants and have beneficial effects on human health (Rao et al., 2018).

### 3.4. Correlation of microorganisms with physicochemical properties and volatile compounds

**Figure 5:**
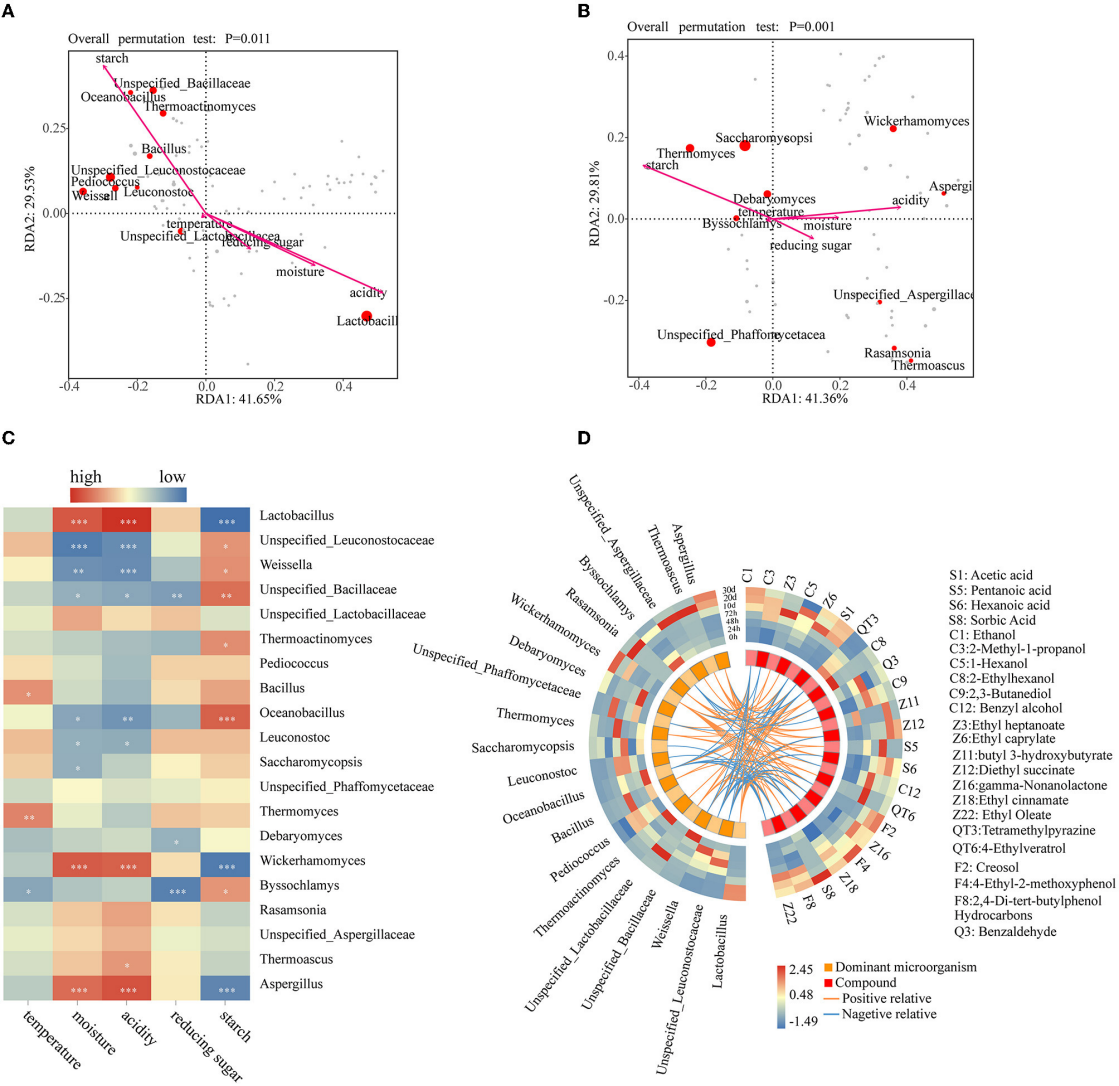
RDA analysis of the physicochemical properties and the bacterial **(A)** and fungal genera **(B)**, the heat map of the correlation index between the top 10 microbial genera in relative abundance and physicochemical properties **(C)**, and circos visualization figure between significantly different volatile compounds and dominant microbial genera in the Xiasha round of the cave brewing sauce-flavor Baijiu based on the Pearson correlation coefficient **(D)** [when the Pearson correlation coefficient is more significant than 0.5, it means that the absolute value of the Pearson correlation coefficient is positively correlated (orange line) or negatively correlated (blue line) with statistically significant differences (*P* < 0.05)].

The effect of physicochemical characteristics on the dominant bacterial and fungal genera was revealed by the redundancy analysis (RDA), with the first two axes explaining 71.18 and 71.17% of the variations in community composition, respectively (Figures 5A, B). As shown in Figure 5C, among the bacterial communities, *Unspecified_Leuconostocaceae, Weissella, Unspecified_Bacillaceae, Oceanobacillus*, and *Thermoactinomyces* were significantly positively correlated with starch but negatively with acidity. Only *Lactobacillus* showed a highly significant negative correlation with starch (*P* < 0.001) but showed a highly significant positive correlation with acidity and moisture (*P* < 0.001). *Bacillus* and *Unspecified_Bacillaceae* were significantly positively and negatively correlated with temperature and reducing sugar, respectively. It shows that high moisture content and acidity can inhibit the growth of other microorganisms. Regarding fungi, *Wickerhamomyces* and *Aspergillus* showed a significant negative correlation with starch (*P* < 0.001) and a positive correlation with moisture and acidity (*P* < 0.001) (Figure 5C). Temperature showed a significant positive correlation and a significant negative correlation with *Thermomyces* and *Byssochlamys*, respectively. Only *Saccharomycopsis* has a negative correlation with moisture. Both *Debaryomyces* and *Byssochlamys* showed a significantly negative correlation with reducing sugar. Only *Byssochlamys* showed a significant positive correlation with starch. *Lactobacillus* and *Aspergillus* had a highly significant positive correlation with moisture and acidity, consistent with the research result of nong sauce–flavored Baijiu in a conventional environment (Zhuansun et al., 2022). They have a highly significant negative correlation with starch. These illustrate that they may all be related to the breakdown of starch. *Aspergillus* is an important filamentous fungus that produces a variety of enzymes to degrade starchy material into fermentable sugars during the fermentation of Chinese Baijiu (Chen et al., 2014). The RDA showed that microorganisms producing amylase have a significant influence on starch in the SF. At the same time, after entering the CF, the monosaccharide produced by starch decomposition provided the conditions for the growth of other microorganisms and influenced the acidity changes. Based on these results, starch was the main physicochemical property factor that affects microbial succession in the SF stage. At the same time, acidity, moisture, and reducing sugar were the main driving factors of microbial succession in the CF stage. This is different from the research results of the traditional brewing environment, in that reducing sugar is the key factor affecting the microbial succession during the SF process of sauce-flavor Baijiu, while acidity, alcoholicity, and temperature are the main factors affecting the microbial succession during the CF process (Hao et al., 2021). Previous research results of light flavor Baijiu showed that acidity, reducing sugar, and temperature were the decisive factors affecting its bacterial succession (Shen et al., 2021). During the fermentation of strong-flavor Baijiu, starch, moisture, and pH significantly affected microbial succession (Guan et al., 2020). These dominant microorganisms were regulated by the physicochemical properties by influencing the physicochemical properties.

Based on Pearson correlation analysis between the dominant microbial genera and the significantly different volatile metabolites visualized (Figure 5D), regarding the bacterial genera, *Lactobacillus* and *Weissella* were the genera with the maximum quantity of positive and negative correlations with 11 and 12 significantly different volatile metabolites, respectively. For fungi, *Wickerhamomyces* and *Aspergillus* were positively associated with 10 significantly different volatile metabolites, respectively, while *Byssochlamys* was negatively associated with four significantly different volatile metabolites.

Acetic acid (S1) and ethanol (C1) were the basis for the formation of various complex compounds, both of which showed significant negative correlations (*P* < 0.05, r < −0.5) with *Weissella, Unspecified_Leuconostocaceae, Unspecified_Bacillaceae*, and *Oceanobacillus* but highly significant positive correlations (*P* < 0.001, r > 0.6) with *Wickerhamomyces, Lactobacillus*, and *Aspergillus*. *Wickerhamomyces* and *Lactobacillus* were positively correlated with 10 and 11 significantly different metabolites, respectively, indicating that they were in the direction of anabolic products under anaerobic conditions with high acidity (Hu X. L. et al., 2021; Lin et al., 2022). *Aspergillus* was positively correlated with 10 significantly different metabolites. *Aspergillus* can secrete various enzymes to convert raw materials into small molecules and supply a material basis for the growth and metabolism of the microbial community (Ali et al., 2018; Zhao G. Z. et al., 2020). To the best of our knowledge, the contents of esters, phenols, alcohols, and acids are higher in CF. Previous studies indicated that *Lactobacillus* and *Aspergillus* vitally contribute to the synthesis of esters (He et al., 2022; Xu et al., 2023). Ethyl caprylate (Z6), diethyl succinate (Z12), gamma-nonalactone (Z16), and ethyl oleate (Z22) showed a positive correlation with *Aspergillus* (*P* < 0.01, r > 0.5). Diethyl succinate (Z12), gamma-nonalactone (Z16), ethyl cinnamate (Z18), and ethyl oleate (Z22) showed a positive correlation with *Lactobacillus* (*P* < 0.01, r > 0.5). These esters were significantly negatively correlated with *Weissella*. Unsurprisingly, this correlation is not consistent with the previous report that *Weissella* has the function of synthesizing esters (Hasan et al., 2006). *Wickerhamomyces* and *Unspecified_Lactobacillaceae* have a significant positive correlation with four esters separately. Compared with the SF stage, these microorganisms with many positive correlations with ester substances have higher relative abundance in the CF stage than in the SF stage. It shows that they have significant contributions to the synthesis of esters. *Wickerhamomyces, Unspecified_Phaffomycetaceae, Unspecified_Lactobacillaceae, Thermoascus, Rasamsonia, Aspergillus*, and *Lactobacillus* were significantly positively correlated with acids (acetic acid, pentanoic acid, hexanoic acid, and sorbic acid). This is consistent with the previous statement that *Aspergillus* and *Lactobacillus* have the ability to produce organic acids (Tang et al., 2019). *Wickerhamomyces, Thermoascus, Rasamsonia, Lactobacillus*, and *Aspergillus* have a significantly positive correlation with alcohols (ethanol, 1-hexanol, 2-ethylhexanol, and benzyl alcohol). *Lactobacillus* can produce ethanol by heterofermentation (Du et al., 2021). The microorganisms with a significant positive correlation with phenols (creosol, 4-ethyl-2-methoxyphenol, and 2,4-di-tert-butylphenol) were *Wickerhamomyces, Unspecified_Lactobacillaceae, Thermoascus, Lactobacillus*, and *Aspergillus*. The research results from a conventional environment showed that phenols are mainly related to *Kroppenstedtia, Staphylococcus*, and *Thermoactinomyces* (Zhuansun et al., 2022). The relative abundance of these microorganisms in the CF stage is higher than that in the SF stage. It shows that the CF stage is the main period for the accumulation of flavor substances, and the production of flavor substances is likely the result of the cooperation of microorganisms and biochemical reactions.

### 3.5. Metabolic mapping of dominant microorganism genera during the fermentation

**Figure 6:**
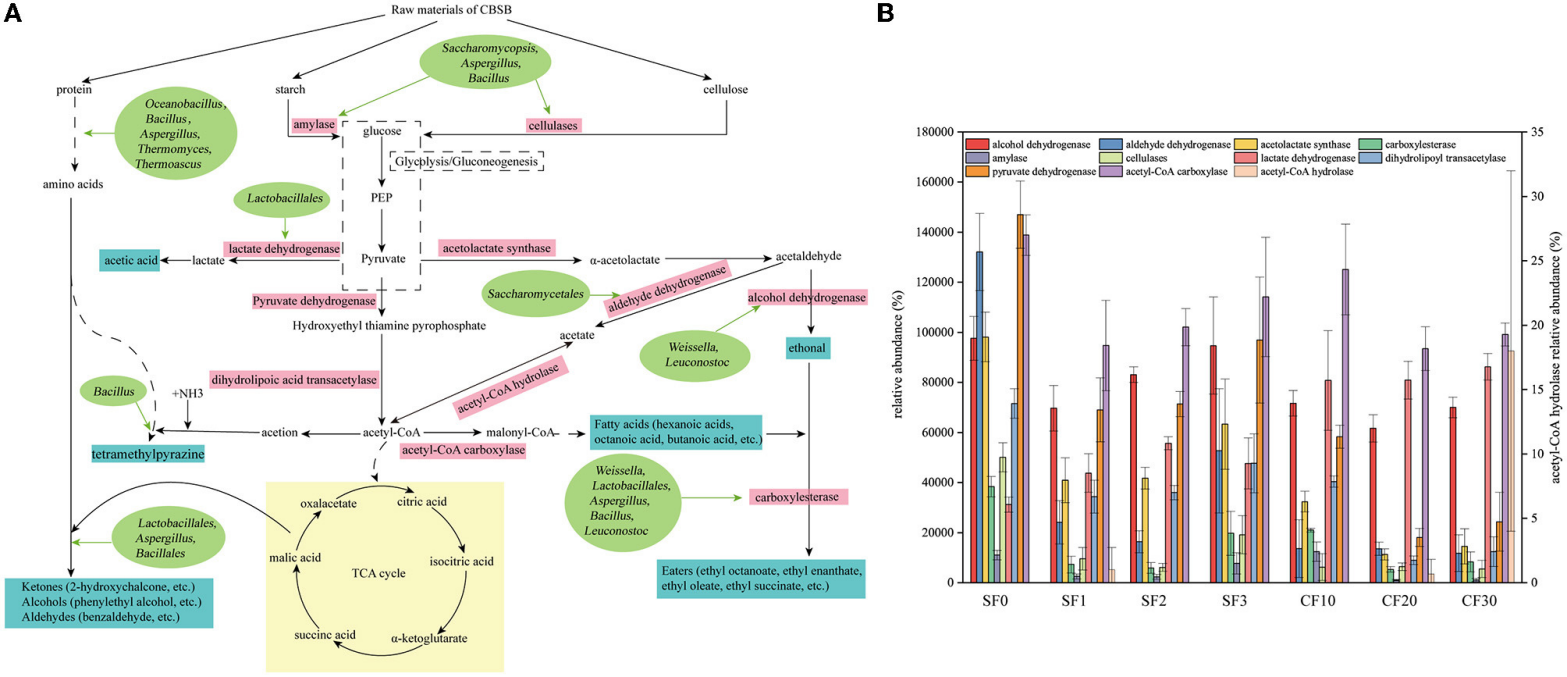
Metabolic profile of the dominant microorganisms **(A)** and relative abundance of enzymes **(B)** during the Xiasha round of the cave brew sauce-flavor Baijiu.

To better understand the relationship between microorganisms and volatile flavors, metabolic mapping of dominant microorganisms during the Xiasha round of the CBSB was performed based on microbial annotated related enzymes and combined with the KEGG database and relevant literature (Figure 6A). Starch and cellulose as raw materials are metabolized by *Saccharomycopsis*, and *Aspergillus* and *Bacillus* secreted amylase and cellulase to form monosaccharides (Gopinath et al., 2017; Jin et al., 2019; Huang et al., 2020). Subsequently, monosaccharides are converted into the critical intermediate metabolite pyruvate, which is involved in the downstream of sugar metabolism. Pyruvate is converted into acetyl-CoA by the action of aldehyde dehydrogenase secreted by *Saccharomycetales*. Acetyl-CoA enters the TCA cycle with the participation of *Lactobacillales, Bacillales*, and *Aspergillus* to form alcohols, aldehydes, and ketones with amino acids. Among them, amino acids are derived from protein degradation by proteases secreted by *Thermoascus, Thermomyces, Aspergillus, Bacillus*, and *Oceanobacillus* (Ali et al., 2018; Jin et al., 2019; Park et al., 2019; Zhao G. Z. et al., 2020). Meanwhile, acetyl-CoA is converted into malonyl-CoA through the action of acetyl-CoA carboxylase to generate fatty acids and then forms an ester with ethanol with the participation of *Weissella, Lactobacillales, Bacillus, Leuconostoc*, and *Aspergillus* (Zhao C. et al., 2020). *Lactobacillale* is the main microorganism responsible for the production of lactic acid, which not only inhibits harmful microorganisms but also acts as a precursor for the formation of ethyl lactate (Jin et al., 2017; Zhang et al., 2018). In addition, *Bacillus* is associated with the synthesis of tetramethylpyrazine (Zhu and Xu, 2010a,b). The changes in the corresponding enzyme abundance are shown in Supplementary Table S5 and Figure 6B. Regarding the raw materials for Baijiu, the three main types of substances initially provided for microbial utilization are starch, cellulose, and protein. The average relative abundance of cellulase in SF was much higher than that in CF and increased significantly on day 3, which might be related to the significantly increased *Bacillus* during this period as it was the primary cellulase producer (Huang et al., 2020). The highest average relative abundance of amylase was observed on day 10 of CF, indicating that the content of starch computation was the largest on day 10, which was consistent with our aforementioned research result that significantly reduced the starch content on day 10 of the CF stage. The relative abundance of lactate dehydrogenase and acetyl CoA hydrolase in CF was higher than in the SF stage. We observed that the acid content increased significantly after entering the CF. Lactate, (S)-isopropyl lactate, and isobutyl lactate also have the same change trend, and *Lactobacillus* was the absolute dominant bacteria in the CF. As shown in Figure 6A, the average relative abundance of microorganisms producing carboxylesterase in CF was greater than that in the SF stage, which was consistent with the significant increase in ester content in the previous CF process.

## 4. Conclusion

We investigated the dynamic changes of a microbial community, physicochemical properties, and volatile flavor compounds during the Xiasha round fermentation process of the CBSB. During the Xiasha round of the CBSB, the SF progress enriched the microflora necessary for flavor formation, and the flavor substance accumulation was mainly concentrated in the CF progress. This study clarified the microbial community structure in the fermentation process of Xiasha round in the CBSB. We revealed the correlation between microbial community, physicochemical properties, and flavor substances and established the correlation networks and metabolic maps to better understand the relationship between dominant microbial genera and critical flavor compounds in the fermentation process. We have demonstrated the potential role of dominant microorganism genera in flavor formation. In this study, most of the dominant microorganisms we detected also appeared in the traditional environmental fermentation process, which fully demonstrated the feasibility of brewing sauce-flavor Baijiu in the natural caves. Thus, these findings will provide theoretical bases for CBSB and thus lay a solid foundation for craft optimization and Baijiu quality improvement. Meanwhile, we are providing technical support for the further development of microbial resources for Baijiu brewing.

## Data availability statement

The data presented in the study are deposited in the National Center for Biotechnology Information (NCBI) repository, accession number of bacteria and fungi are PRJNA928402 and PRJNA928447, respectively.

## Author contributions

TTR and WS contributed the experimental design. TTR performed the statistical analysis and wrote the manuscript. WS and DWZ provided expertise and insight relating to Baijiu microbiology. TTR, WS, YCM, and QQ contributed to manuscript revision, read, and approved the submitted version. All authors contributed to the article and approved the submitted version.
