# Supplementary material for: Study on the correlation between microbial communities with physicochemical properties and flavor substances in the Xiasha round of cave-brewed sauce-flavor Baijiu
Source: Front Microbiol. 2023 Mar 1;14:1124817. doi: 10.3389/fmicb.2023.1124817 (PMC10014610; doi:10.3389/fmicb.2023.1124817)
Supplement: Supplementary file 1 [file Data_Sheet_1.pdf]

Supplementary Material

Study on the correlation between microbial communities with physicochemical properties and flavor substances in the Xiasha round of cave brewed Sauce-flavor Baijiu

Tingting Ren[1, 2], Wei Su[1,2*], Yingchun Mu[1], Qi Qi[1] and Dangwei Zhang[1,2]

[1] School of Liquor and Food Engineering, Guizhou University, Guiyang 550025, China;

[2] Guizhou Provincial Key Laboratory of Fermentation Engineering and Biological Pharmacy Guizhou University, Guiyang 550025, China.

*** The complete contact details of the corresponding author:**

Name: Wei Su;

E-mail: suwei1886@163.com;

Phone: +0851-3621956;

Fax: +0851-3621956;

Mailing address: School of Liquor and Food Engineering, Guizhou University, Guiyang 550025, China.

The number of supplementary figures: 2

The number of supplementary tables: 5

The order of the supplementary figures and supplementary tables is the same order as that in the manuscript.

Table S1 Operating units and sequence abundance of bacteria and fungi in different fermentation stages in the Xiasha round of the cave-brewed sauce-flavor Baijiu

Sample ID	Bacteria		Fungi	
	non-chimeric	percentage of input non-chimeric	Non-chimeric	Percentage of input non-chumeric
SF0-1	38841	78.07	22150	62.57
SF0-2	35011	75.29	22682	59.78
SF0-3	35441	78.06	21272	64.27
SF1-1	25651	77.99	24389	74.15
SF1-2	25917	71.68	29113	75.8
SF1-3	34063	78.94	27935	78.26
SF2-1	29653	69.21	39450	83.53
SF2-2	30594	77.13	32257	82.14
SF2-3	32736	81.34	36605	84.26
SF3-1	32990	73.77	27606	66.65
SF3-2	36555	81.16	28951	65.24
SF3-3	25913	59.69	24709	67.69
CF10-1	23824	53.18	16487	42.72
CF10-2	26773	66.16	26979	53.99
CF10-3	25426	63.96	25512	48.71
CF20-1	33034	86.08	21905	50.25
CF20-2	29275	81.62	29905	73.16
CF20-3	27278	76.04	22264	48.03
CF30-1	31988	83.65	29872	65.53
CF30-2	30246	73.31	26873	61.15
CF30-3	32818	78.43	24428	64.28

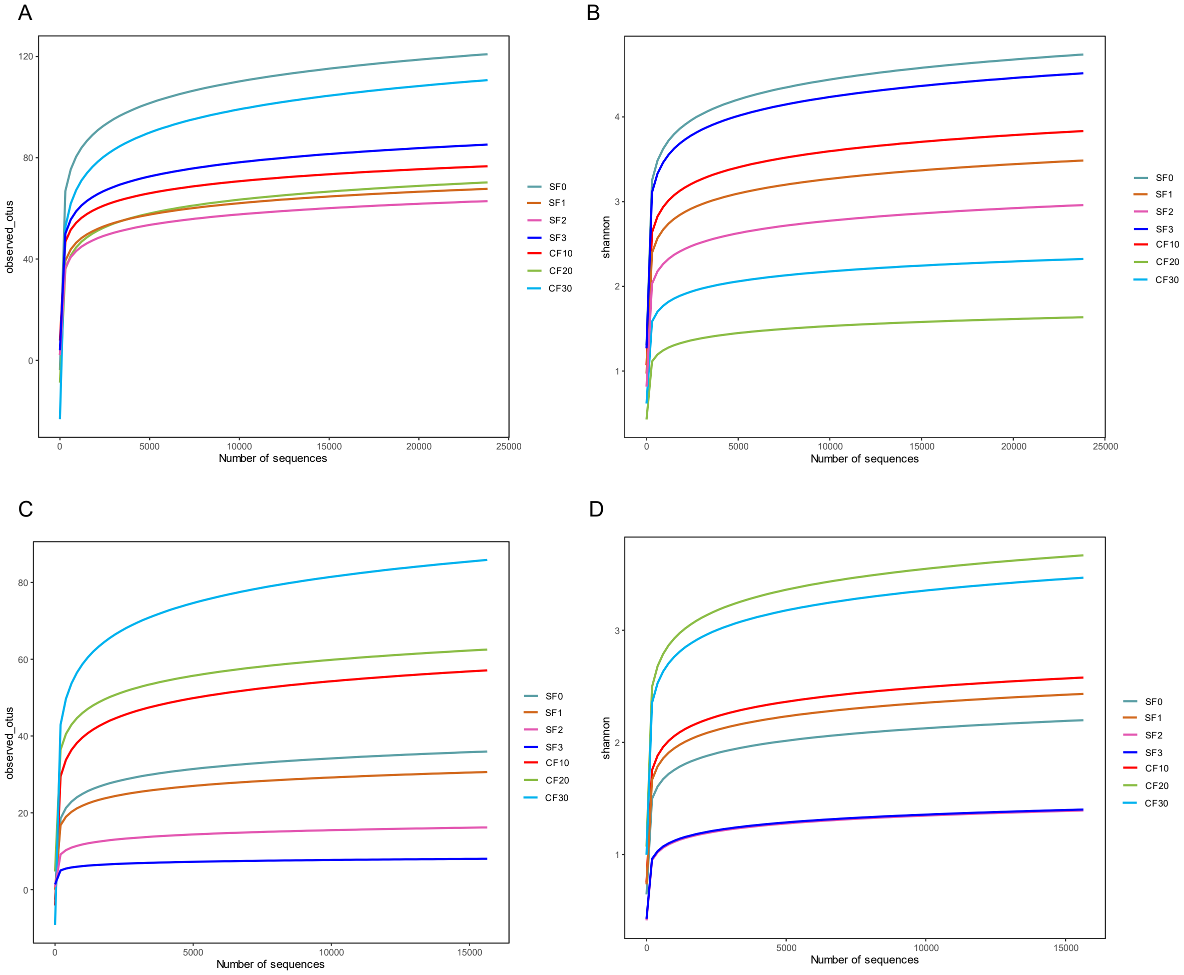

Fig. S1 Dilution curve and shannon curve of bacteria and fungi in different fermentation stages in the Xiasha round of the cave-brewed sauce-flavor Baijiu

Table S2 Diversity indices during fermentation in the Xiasha round of the cave-brewed sauce-flavor Baijiu

Samples	chao1		shannon		simpson	
	Bacteria	fungi	Bacteria	fungi	Bacteria	fungi
SF0	119.67 ± 11.15[a]	35.33 ± 3.21[bc]	4.46 ± 0.12[a]	2.07 ± 0.13[bc]	0.90 ± 0.01[a]	0.65 ± 0.00[ab]
SF1	65.67 ± 16.92[b]	30.00 ± 8.89[bc]	3.28 ± 0.51[abc]	2.29 ± 0.57[b]	0.80 ± 0.06[a]	0.69 ± 0.10[ab]
SF2	61.17 ± 14.49[b]	16.00 ± 1.73[c]	2.78 ± 0.64[bc]	1.31 ± 0.36[c]	0.66 ± 0.14[a]	0.43 ± 0.12[c]
SF3	83.00 ± 15.87[ab]	8.00 ± 2.00[c]	4.25 ± 0.64[a]	1.32 ± 0.45[c]	0.88 ± 0.08[a]	0.52 ± 0.14[bc]
CF10	73.50 ± 10.40[b]	56.00 ± 27.62[ab]	3.60 ± 0.76[ab]	2.42 ± 0.46[b]	0.78 ± 0.12[a]	0.63 ± 0.04[ab]
CF20	70.67 ± 27.15[b]	59.67 ± 8.02[ab]	1.54 ± 0.16[d]	3.45 ± 0.06[a]	0.33 ± 0.04[b]	0.82 ± 0.03[a]
CF30	112.17 ± 19.28[a]	84.67 ± 31.56[a]	2.19 ± 0.66[cd]	3.26 ± 0.09[a]	0.48 ± 0.14[b]	0.80 ± 0.01[a]

Note: Data are presented as means ± standard deviations (n = 2). Different letters within a column are significantly different statistically ($P < 0.05$).

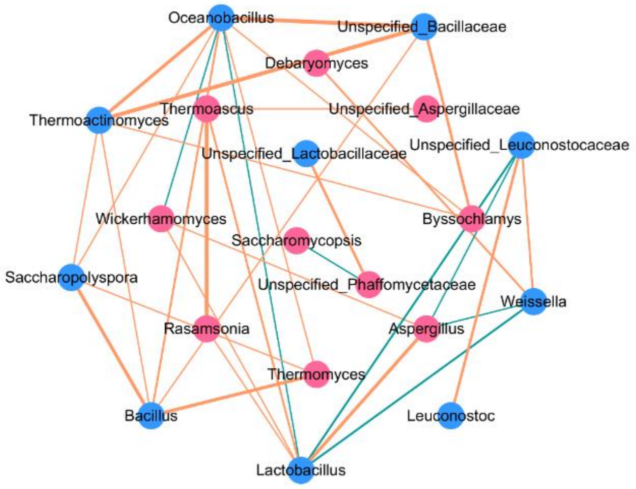

Fig. S2 Fungal-bacterial association network diagram, red circles and blue circles indicate fungi and bacteria respectively, orange lines and green lines indicate positive ($P < 0.05$, $r > 0.5$) and negative ($P < 0.05$, $r < -0.5$) correlations, respectively

Table S3 Changes of volatile flavor compounds in different fermentation stages

Code	RT	Compound	Content (μg/Kg)						
			SF0	SF1	SF2	SF3	CF10	CF20	CF30
Z1	8.33	**Isoamyl acetate**	n.d.	n.d.	1.07 ± 0.27^{c}	3.12 ± 0.56^{b}	4.67 ± 0.46^{a}	n.d.	0.59 ± 0.05^{cd}
Z2	11.77	**Ethyl Hexanoate**	0.74 ± 0.13^{c}	0.96 ± 0.35^{c}	3.73 ± 0.51^{c}	9.49 ± 2.53^{b}	15.44 ± 2.39^{a}	13.70 ± 1.80^{a}	7.30 ± 0.40^{b}
Z3	14.70	**Ethyl heptanoate**	n.d.	n.d.	0.07 ± 0.01^{bc}	0.40 ± 0.24^{b}	1.58 ± 0.27^{a}	0.21 ± 0.09^{bc}	0.23 ± 0.04^{bc}
Z4	14.97	**Lactate**	2.18 ± 0.12^{d}	1.84 ± 0.06^{d}	1.90 ± 0.15^{d}	2.81 ± 0.30^{d}	29.59 ± 2.11^{c}	64.36 ± 0.90^{b}	95.20 ± 0.64^{a}
Z5	17.22	**(S)-Isopropyl lactate**	n.d.	n.d.	n.d.	n.d.	5.53 ± 0.44^{a}	1.65 ± 0.06^{b}	1.45 ± 0.12^{b}
Z6	17.45	**Ethyl caprylate**	n.d.	0.05 ± 0.02^{d}	0.24 ± 0.08^{d}	0.72 ± 0.21^{d}	3.69 ± 0.58^{a}	1.46 ± 0.30^{c}	2.16 ± 0.45^{b}
Z7	18.07	**Isobutyl lactate**	n.d.	n.d.	n.d.	n.d.	n.d.	0.10 ± 0.02^{b}	0.29 ± 0.07^{a}
Z8	20.20	**Ethyl 2-hydroxy-4-methylvalerate**	n.d.	n.d.	n.d.	n.d.	5.59 ± 0.87^{b}	n.d.	18.86 ± 1.17^{a}
Z9	21.82	**Spironolactone**	n.d.	1.51 ± 0.37^{ab}	0.47 ± 0.17^{b}	0.93 ± 0.32^{ab}	1.41 ± 0.42^{ab}	2.73 ± 0.61^{a}	0.66 ± 0.19^{ab}
Z10	22.47	**Ethyl caprate**	n.d.	n.d.	n.d.	0.10 ± 0.03^{b}	0.51 ± 0.08^{a}	n.d.	0.48 ± 0.11^{a}
Z11	22.53	**Butyl 3-hydroxybutyrate**	0.41 ± 0.05^{b}	3.71 ± 0.80^{a}	n.d.	n.d.	n.d.	4.42 ± 0.72^{a}	0.41 ± 0.17^{b}
Z12	23.43	**Diethyl succinate**	0.53 ± 0.06^{b}	0.30 ± 0.04^{b}	1.75 ± 0.65^{b}	3.20 ± 0.62^{b}	3.25 ± 0.81^{b}	10.45 ± 0.78^{a}	8.37 ± 0.61^{a}
Z13	25.82	**Ethyl phenylacetate**	0.22 ± 0.02^{e}	0.31 ± 0.01^{e}	0.37 ± 0.14^{e}	1.94 ± 0.65^{d}	4.85 ± 0.69^{b}	3.70 ± 0.50^{c}	6.44 ± 0.75^{a}
Z14	26.45	**Phenethyl acetate**	0.40 ± 0.07^{d}	0.77 ± 0.11^{d}	5.19 ± 0.67^{c}	16.08 ± 3.00^{b}	24.51 ± 2.67^{a}	4.16 ± 0.37^{c}	4.74 ± 0.59^{c}
Z15	27.92	**Ethyl 3-phenylpropionate**	n.d.	0.09 ± 0.01^{c}	0.74 ± 0.21^{b}	n.d.	3.27 ± 0.68^{a}	1.13 ± 0.15^{b}	n.d.
Z16	31.76	**gamma-Nonanolactone**	0.18 ± 0.01^{cd}	0.08 ± 0.02^{d}	0.22 ± 0.05^{cd}	0.26 ± 0.08^{c}	0.57 ± 0.12^{a}	0.42 ± 0.06^{b}	0.41 ± 0.03^{b}
Z17	32.33	**Ethyl myristate**	n.d.	n.d.	0.01 ± 0.00^{b}	0.05 ± 0.01^{b}	1.62 ± 0.14^{a}	1.65 ± 0.15^{a}	1.56 ± 0.21^{a}
Z18	34.33	**Ethyl cinnamate**	n.d.	n.d.	0.12 ± 0.01^{b}	0.17 ± 0.06^{ab}	0.21 ± 0.04^{a}	0.15 ± 0.02^{ab}	0.20 ± 0.04^{ab}
Z19	37.09	**Palmitic acid ethyl ester**	2.00 ± 0.32^{b}	1.98 ± 0.62^{b}	1.70 ± 0.17^{b}	3.69 ± 0.64^{b}	41.13 ± 1.99^{a}	0.73 ± 0.07^{b}	41.03 ± 6.38^{a}
Z20	37.60	**Ethyl 9-hexadecenoate**	0.01 ± 0.00^{d}	n.d.	0.03 ± 0.01^{d}	0.11 ± 0.05^{d}	1.8 ± 0.11^{a}	0.92 ± 0.11^{b}	0.74 ± 0.13^{c}
Z21	38.01	**Dimethyl phthalate**	n.d.	0.02 ± 0.01^{b}	n.d.	n.d.	n.d.	0.11 ± 0.01^{a}	n.d.
Z22	41.34	**Ethyl Oleate**	0.64 ± 0.11^{d}	0.81 ± 0.23^{d}	0.60 ± 0.03^{d}	1.64 ± 0.22^{d}	10.51 ± 0.52^{a}	7.95 ± 0.94^{b}	6.70 ± 0.22^{c}
Z23	42.16	**Mandenol**	0.73 ± 0.16^{c}	0.88 ± 0.24^{c}	0.69 ± 0.03^{c}	2.30 ± 0.32^{c}	15.99 ± 0.65^{a}	14.57 ± 1.41^{a}	11.57 ± 2.16^{b}
C1	3.53	**Ethanol**	n.d.	0.01 ± 0.00^{d}	3.40 ± 0.57^{d}	11.94 ± 1.01^{c}	42.25 ± 5.18^{b}	54.72 ± 4.36^{a}	56.77 ± 3.36^{a}
C2	5.34	**2-Butanol**	n.d.	n.d.	n.d.	n.d.	0.57 ± 0.13^{a}	0.40 ± 0.09^{b}	n.d.
C3	7.32	**2-Methyl-1-propanol**	n.d.	n.d.	0.39 ± 0.05^{c}	1.40 ± 0.49^{a}	1.40 ± 0.17^{a}	1.42 ± 0.11^{a}	0.83 ± 0.07^{b}
C4	10.93	**3-Methyl-1-butanol**	1.53 ± 0.23^{f}	3.59 ± 0.31^{f}	23.05 ± 1.99^{e}	32.26 ± 1.62^{d}	58.03 ± 4.30^{a}	44.95 ± 3.03^{b}	40.12 ± 0.52^{c}
C5	15.21	**1-Hexanol**	2.26 ± 0.04^{cd}	2.80 ± 0.27^{bc}	3.14 ± 0.12^{bc}	3.78 ± 0.20^{b}	3.75 ± 0.51^{b}	5.28 ± 0.94^{a}	1.80 ± 0.11^{d}
C6	17.04	**(S)-2-octano**	n.d.	n.d.	0.78 ± 0.12^{a}	n.d.	n.d.	n.d.	0.16 ± 0.05^{a}
C7	17.85	**1-Octen-3-ol**	0.05 ± 0.01^{c}	n.d.	0.40 ± 0.02^{a}	0.28 ± 0.08^{b}	n.d.	n.d.	n.d.
C8	18.85	**2-Ethylhexanol**	36.60 ± 14.52^{a}	n.d.	1.24 ± 0.16^{a}	0.70 ± 0.02^{a}	0.45 ± 0.09^{a}	n.d.	5.31 ± 0.92^{a}
C9	21.04	**2,3-Butanediol**	n.d.	n.d.	0.73 ± 0.14^{b}	1.02 ± 0.20^{b}	1.63 ± 0.39^{a}	1.16 ± 0.04^{b}	0.82 ± 0.13^{b}
C10	22.95	**1-Nonanol**	0.09 ± 0.03^{b}	0.30 ± 0.09^{b}	0.07 ± 0.01^{b}	0.18 ± 0.04^{b}	0.54 ± 0.08^{a}	0.23 ± 0.01^{b}	n.d.
C11	23.10	**2-Furanmethanol**	1.30 ± 0.15^{bc}	1.69 ± 0.04^{bc}	3.03 ± 0.46^{ab}	2.54 ± 0.21^{ab}	n.d.	3.11 ± 0.30^{ab}	4.14 ± 0.45^{a}
C12	27.79	**Benzyl alcohol**	0.49 ± 0.07^{c}	0.43 ± 0.02^{c}	2.31 ± 0.20^{b}	4.99 ± 0.40^{a}	2.26 ± 0.20^{b}	2.05 ± 0.14^{b}	2.50 ± 0.11^{b}
C13	28.59	**Phenylethyl Alcohol**	n.d.	n.d.	61.48 ± 7.09^{d}	100.53 ± 8.89^{b}	127.53 ± 6.45^{a}	88.47 ± 3.90^{c}	96.59 ± 11.49^{bc}
S1	17.94	**Acetic acid**	3.20 ± 0.30^{d}	3.75 ± 0.11^{d}	4.06 ± 0.14^{d}	9.49 ± 1.22^{c}	26.55 ± 1.07^{a}	23.57 ± 1.69^{b}	24.47 ± 1.28^{b}
S2	20.13	**Propanoic acid**	n.d.	n.d.	n.d.	n.d.	4.53 ± 0.58	n.d.	n.d.
S3	22.26	**Butanoic acid**	1.29 ± 0.11^{c}	1.65 ± 0.31^{c}	2.83 ± 0.17^{c}	3.96 ± 0.90^{c}	5.22 ± 0.35^{c}	17.05 ± 0.50^{b}	58.64 ± 5.65^{a}

S4	23.20	**Isovaleric acid**	4.04 ± 0.19^{e}	3.13 ± 0.21^{e}	8.22 ± 0.87^{d}	10.14 ± 1.23^{c}	11.48 ± 0.32^{b}	13.39 ± 0.38^{a}	13.45 ± 0.70^{a}
S5	24.75	**Pentanoic acid**	n.d.	n.d.	0.88 ± 0.79^{a}	n.d.	2.08 ± 1.21^{a}	n.d.	n.d.
S6	27.06	**Hexanoic acid**	5.30 ± 0.19^{d}	3.88 ± 0.60^{d}	6.17 ± 0.87^{d}	13.13 ± 3.79^{c}	16.98 ± 1.14^{b}	20.62 ± 1.89^{a}	14.70 ± 0.78^{bc}
S7	32.64	**Octanoic acid**	0.07 ± 0.02^{d}	0.03 ± 0.02^{d}	0.13 ± 0.01^{d}	0.40 ± 0.16^{c}	1.05 ± 0.30^{a}	0.53 ± 0.10^{bc}	0.73 ± 0.08^{b}
S8	34.80	**Sorbic Acid**	1.29 ± 0.03^{b}	n.d.	n.d.	n.d.	n.d.	n.d.	10.73 ± 2.17^{a}
S9	40.54	**Benzoic acid**	n.d.	n.d.	n.d.	0.05 ± 0.02^{c}	0.24 ± 0.13^{b}	0.17 ± 0.05^{b}	0.50 ± 0.07^{a}
S10	42.76	**Benzeneacetic acid**	n.d.	n.d.	n.d.	0.12 ± 0.03^{c}	0.30 ± 0.10^{b}	0.31 ± 0.08^{b}	0.88 ± 0.09^{a}
S11	48.11	**Palmitic acid**	0.45 ± 0.02^{c}	0.16 ± 0.03^{c}	0.28 ± 0.04^{c}	0.82 ± 0.10^{bc}	0.88 ± 0.16^{bc}	1.49 ± 0.42^{ab}	2.03 ± 0.41^{a}
F1	27.44	**Guaiacol**	0.41 ± 0.04^{cd}	0.31 ± 0.02^{d}	0.59 ± 0.09^{c}	0.51 ± 0.08^{cd}	0.44 ± 0.04^{cd}	1.55 ± 0.13^{b}	1.81 ± 0.16^{a}
F2	29.85	**Creosol**	n.d.	n.d.	0.05 ± 0.01^{d}	0.51 ± 0.12^{d}	5.43 ± 0.61^{c}	12.30 ± 0.98^{b}	14.58 ± 1.88^{a}
F3	31.29	**Phenol**	0.94 ± 0.13^{d}	0.75 ± 0.08^{d}	1.38 ± 0.11^{c}	1.76 ± 0.15^{c}	1.70 ± 0.18^{c}	4.34 ± 0.26^{b}	4.84 ± 0.41^{a}
F4	31.88	**4-Ethyl-2-methoxyphenol**	0.13 ± 0.06^{d}	0.37 ± 0.14^{d}	5.87 ± 0.84^{cd}	11.26 ± 1.59^{c}	31.52 ± 3.64^{b}	36.74 ± 2.33^{b}	72.98 ± 7.73^{a}
F5	33.28	**p-Cresol**	0.02 ± 0.01^{b}	n.d.	0.04 ± 0.01^{b}	0.09 ± 0.03^{b}	0.21 ± 0.09^{a}	0.09 ± 0.02^{b}	0.06 ± 0.03^{b}
F6	35.46	**4-Ethylphenol**	0.06 ± 0.01^{f}	0.10 ± 0.02^{f}	1.17 ± 0.13^{e}	2.21 ± 0.33^{d}	4.98 ± 0.66^{c}	6.24 ± 0.57^{b}	9.03 ± 1.11^{a}
F7	35.92	**2-Methoxy-4-vinylphenol**	0.84 ± 0.16^{a}	0.52 ± 0.06^{b}	0.29 ± 0.04^{c}	0.28 ± 0.10^{c}	n.d.	n.d.	0.06 ± 0.05^{d}
F8	38.38	**2,4-Di-tert-butylphenol**	10.92 ± 1.16^{cd}	14.77 ± 3.26^{c}	6.13 ± 1.32^{d}	7.36 ± 1.83^{d}	33.95 ± 1.89^{a}	26.88 ± 1.86^{b}	31.08 ± 3.49^{a}b
Q1	16.34	**Nonanal**	n.d.	n.d.	0.88 ± 0.40^{a}	1.06 ± 0.94^{a}	1.15 ± 1.00^{a}	n.d.	n.d.
Q2	19.08	**Decanal**	0.11 ± 0.10^{a}	n.d.	0.53 ± 0.51^{a}	n.d.	n.d.	n.d.	n.d.
Q3	19.73	**Benzaldehyde**	2.49 ± 0.27^{cd}	1.84 ± 0.18^{d}	3.35 ± 0.02^{b}	5.05 ± 0.73^{a}	1.11 ± 0.10^{e}	2.19 ± 0.31^{d}	2.95 ± 0.20^{bc}
Q4	22.63	**Benzeneacetaldehyde**	1.75 ± 0.15^{ab}	1.46 ± 0.30^{b}	2.01 ± 0.05^{a}	1.67 ± 0.38^{ab}	0.14 ± 0.03^{c}	0.36 ± 0.05^{c}	0.17 ± 0.03^{c}
T1	7.68	**Undecane**	1.20 ± 0.08^{a}	0.29 ± 0.04^{b}	0.07 ± 0.02^{b}	0.03 ± 0.01^{b}	0.19 ± 0.07^{b}	n.d.	0.06 ± 0.01^{b}
T2	13.82	**Tridecane**	n.d.	0.67 ± 0.14^{a}	n.d.	n.d.	0.79 ± 0.17^{a}	0.63 ± 0.06^{a}	n.d.
T3	19.18	**Pentadecane**	0.70 ± 0.29^{a}	0.39 ± 0.07^{a}	n.d.	n.d.	n.d.	1.05 ± 0.09^{a}	1.37 ± 0.14^{a}
T4	20.53	**1-cyclopropylpentane**	0.21 ± 0.02^{bc}	0.37 ± 0.11^{b}	0.45 ± 0.12^{b}	0.44 ± 0.05^{b}	0.82 ± 0.05^{a}	0.28 ± 0.03^{bc}	n.d.
T5	12.44	**Styrene**	0.19 ± 0.09^{c}	0.33 ± 0.05^{c}	1.05 ± 0.37^{bc}	0.78 ± 0.23^{bc}	1.73 ± 0.23^{b}	1.34 ± 0.09^{b}	2.74 ± 0.10^{a}
T6	32.18	**3,4-Dimethoxystyrol**	0.01 ± 0.00^{b}	n.d.	0.09 ± 0.02^{b}	0.94 ± 0.14^{a}	n.d.	n.d.	n.d.
QT1	3.07	**2-Hydroxychalcone**	0.03 ± 0.01	n.d.	n.d.	n.d.	n.d.	n.d.	n.d.
QT2	4.32	**Dimethyl ether**	n.d.	n.d.	n.d.	n.d.	n.d.	3.66 ± 0.12	n.d.
QT3	18.37	**Tetramethylpyrazine**	9.38 ± 0.63^{a}	6.91 ± 0.37^{b}	6.62 ± 1.19^{b}	4.51 ± 0.49^{c}	1.50 ± 0.39^{d}	1.31 ± 0.13^{d}	1.39 ± 0.12^{d}
QT4	21.99	**2-Acetyl-5-methylfuran**	0.44 ± 0.03^{c}	0.39 ± 0.06^{c}	0.74 ± 0.08^{b}	1.00 ± 0.08^{b}	1.25 ± 0.27^{a}	0.51 ± 0.16^{c}	0.79 ± 0.04^{b}
QT5	24.59	**1,2-Dimethoxybenzene**	1.66 ± 0.17^{b}	1.30 ± 0.01^{bc}	1.85 ± 0.61^{b}	3.01 ± 0.45^{a}	0.70 ± 0.12^{cd}	0.45 ± 0.07^{d}	0.70 ± 0.09^{cd}
QT6	27.95	**4-Ethylveratrol**	0.12 ± 0.01^{b}	n.d.	n.d.	7.46 ± 1.87^{a}	n.d.	n.d.	1.45 ± 0.13^{b}
QT7	39.91	**2,3-Dihydrobenzofuran**	0.18 ± 0.03^{a}	0.13 ± 0.01^{a}	0.15 ± 0.01^{a}	0.13 ± 0.06^{a}	n.d.	n.d.	n.d.

Note: n.d. is represent not detected. Data are presented as means ± standard deviations (n = 2). Different letters within a row are significantly different statistically (*P* < 0.05)

Table S4 Total content corresponding to each category of compound

Samples	esters	alcohols	acids	phenols	aldehydes	hydrocarbons	other
SF0	8.04 ± 0.83^{e}	42.33 ± 44.51^{e}	15.64 ± 2.61^{d}	13.26 ± 0.92^{d}	4.35 ± 0.37^{b}	2.31 ± 1.07^{ab}	11.81 ± 0.81^{b}
SF1	13.30 ± 0.69^{e}	8.82 ± 1.22^{e}	12.60 ± 1.04^{d}	16.82 ± 3.35^{d}	3.30 ± 0.23^{b}	2.06 ± 0.96^{ab}	8.72 ± 0.35^{c}
SF2	18.39 ± 2.77^{e}	100.14 ± 6.89^{d}	22.56 ± 2.15^{d}	15.52 ± 2.53^{d}	6.77 ± 0.25^{a}	0.62 ± 0.36^{b}	9.36 ± 1.86^{bc}
SF3	46.85 ± 4.73^{d}	160.83 ± 12.52^{c}	38.11 ± 5.89^{c}	23.98 ± 5.22^{d}	7.77 ± 2.02^{a}	0.94 ± 0.52^{b}	16.10 ± 2.87^{a}
CF10	175.72 ± 18.30^{c}	238.41 ± 15.99^{a}	69.31 ± 2.57^{b}	78.23 ± 6.68^{c}	2.40 ± 1.22^{b}	3.52 ± 0.65^{a}	3.45 ± 0.72^{d}
CF20	134.57 ± 4.79^{b}	201.79 ± 11.03^{b}	77.53 ± 3.84^{b}	88.14 ± 6.08^{b}	2.55 ± 0.37^{b}	3.30 ± 1.14^{a}	5.93 ± 0.95^{d}
CF30	209.39 ± 13.18^{a}	112.46 ± 4.91^{d}	126.15 ± 13.78^{a}	134.42 ± 8.58^{a}	3.12 ± 0.19^{b}	4.18 ± 1.43^{a}	4.34 ± 0.33^{d}

Note: Data are presented as means ± standard deviations (n=2). Different letters within a column are significantly different statistically (*P* < 0.05).

Table S5 Relative abundance of enzyme in sample

Samples	**SF0**	**SF1**	**SF2**	**SF3**	**CF10**	**CF20**	**CF30**
Alcohol dehydrogenase	97626.67 ± 8771.36^{a}	69704.33 ± 9129.00^{b}	83125.67 ± 3110.82^{ab}	94725.33 ± 19405.91^{a}	71665.33 ± 5130.98^{b}	61666.33 ± 5456.30^{b}	70001.33 ± 4162.58^{b}
Aldehyde dehydrogenase	132123.33 ± 15439.83^{a}	24133.67 ± 8675.56^{c}	16420.67 ± 4392.63^{c}	52731.33 ± 24787.81^{b}	13615.67 ± 11497.98^{c}	13528.00 ± 2682.25^{c}	11795.00 ± 7356.76^{c}
Acetolactate synthase	98164.33 ± 9937.17^{a}	40927.33 ± 8925.12^{c}	41743.33 ± 4354.19^{c}	63361.00 ± 18015.97^{b}	32390.00 ± 4125.04^{c}	11282.67 ± 2251.08^{d}	14576.67 ± 7056.74^{d}
Carboxylesterase	38400.67 ± 4071.94^{a}	7280.67 ± 3297.23^{c}	5908.67 ± 2151.99^{c}	19720.67 ± 8824.99^{b}	21026.33 ± 768.03^{b}	5323.00 ± 1071.80^{c}	8382.33 ± 3975.42^{c}
amylase	11088.33 ± 1886.12^{a}	2508.67 ± 963.86^{b}	2364.67 ± 981.36^{b}	7758.67 ± 4257.32^{a}	12399.00 ± 3890.20^{a}	1002.67 ± 274.88^{b}	1113.33 ± 590.70^{b}
Cellulase	50100.67 ± 5807.67^{a}	9600.67 ± 4481.78^{c}	6157.33 ± 1587.44^{c}	19134.00 ± 7622.83^{b}	6215.67 ± 5344.35^{c}	6398.67 ± 1521.47^{c}	5539.67 ± 3421.90^{c}
lactate dehydrogenase	31236.67 ± 2996.91^{c}	43788.67 ± 7688.68^{bc}	55710.67 ± 2600.96^{b}	47645.33 ± 10158.96^{bc}	80821.33 ± 19914.15^{a}	80949.00 ± 7567.21^{a}	86270.00 ± 5291.08^{a}
dihydrolipoic acid transacetylase	71536.33 ± 5916.30^{a}	34363.33 ± 6588.50^{b}	36010.33 ± 2886.10^{b}	47705.67 ± 11792.16^{b}	40439.33 ± 2267.18^{b}	8968.00 ± 1711.16^{c}	12401.33 ± 5887.47^{c}
Pyruvate dehydrogenase	147018.33 ± 13475.70^{a}	69034.00 ± 12773.87^{c}	71346.67 ± 4999.17^{c}	96955.33 ± 25159.98^{b}	58365.33 ± 4585.36^{c}	18115.33 ± 3558.60^{d}	24382.33 ± 11727.16^{d}
Acetyl-CoA hydrolase	n.d.	1.00 ± 1.73^{b}	n.d.	n.d.	n.d.	0.67 ± 1.15^{b}	18.00 ± 14.00^{a}
Acetyl-CoA carboxylase	138823.67 ± 8086.84^{a}	94743.33 ± 18093.91^{b}	102066.00 ± 7374.50^{b}	114149.33 ± 23840.18^{ab}	125123.67 ± 18095.37^{ab}	93449.67 ± 8781.63^{b}	99208.00 ± 4576.20^{b}

Note: Data are presented as means ± standard deviations (n = 2). Different letters within a row are significantly different statistically (*P* < 0.05)
